# Displacement of the Spleen Mimicking Renal Cell Cancer Recurrence Post-Nephrectomy: A Case Report

**DOI:** 10.15586/jkcvhl.2015.36

**Published:** 2015-06-20

**Authors:** Carolina S. Emanuels, Krista D. Timmerman, Tabish Aijaz, Thu-Cuc Nguyen, Nathaniel Jest, Walter E. Drane, Scott M. Gilbert, Paul L. Crispen, Li-Ming Su, Lori A. Deitte, Long H. Dang

**Affiliations:** 1 Division of Hematology/Oncology, Department of Internal Medicine, University of Florida Shands Cancer Center; 2 Department of Radiology; 3 Department of Urology, University of Florida, Gainesville, Florida, USA.

## Abstract

Local regional recurrence of renal cell cancer post-nephrectomy most often occurs within three years after surgery. Post-nephrectomy, many processes may mimic RCC recurrence. We present the case of a 75 year-old Caucasian male patient with a mass in his renal fossa post-nephrectomy for renal cell cancer, suggesting local recurrence. Use of the technetium-99m sulfur colloid scan showed that the mass was his spleen which had been displaced into the renal fossa. With high index of suspicion, characterization of these processes as splenic in origin would prevent subjecting patients to risks of biopsy or even surgery.

## Introduction

It is estimated that 63,920 new cases of kidney and renal pelvis cancer were diagnosed in the United States in 2014, and 13,860 patients succumbed to the disease (1). The incidence of renal cancer has risen by 2–3% every year since the early 1990s, many discovered serendipitously due to the increased use of computed tomography (CT) scans. Of the renal malignancies, 92% are renal cell carcinoma (RCC) (2). RCC, which are adenocarcinomas of the renal tubular epithelium, can be further distinguished into clear cell, (accounting for 70–80% of all RCC), papillary (10–15%), chromophobe and collecting duct carcinoma (6% or less, collectively) (2). Surgery (radical or partial nephrectomy) remains the mainstay of treatment for RCC.

Unfortunately, about one third of patients who undergo surgical resection for localized disease have recurrence, either locally or with distant metastatic disease (1). Therefore active surveillance with regular CT scan is recommended in the first five years after resection (3). Here, we present a unique case of spleen appearing as recurrent tumor after migrating in the surgical defect created by total nephrectomy. To our knowledge, no similar case of complete displacement of spleen in the renal fossa has been reported previously.

## Consent

Written informed consent was obtained from the patient for publication of this case report and accompanying images. A copy of the written consent is available for review.

## Case presentation

**Figure 1.**
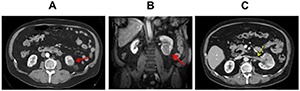
**A.** Axial postcontrast CT image demonstrating the 2 cm cortical based mass in the inferior left kidney (red arrow). **B.** Coronal postcontrast fat-suppressed T1-weighted MR image demonstrating the 4.5 cm infiltrative mass in the inferior left kidney. **C.** Demonstrates a filling defect in the left renal vein (yellow arrow), representing tumor thrombus, approaching but not quite extending into the IVC.

We present the case of a 75 year-old Caucasian male patient who presented to our institution for evaluation of a rapidly growing left renal mass. His initial abdominal CT, performed for flank pain, showed an approximately 2 cm cortically based mass in the left kidney **(Figure 1A)**. He presented again after 13 months, at which time he had an abdominal MRI. This again showed the left renal mass, which had grown to 4.5 cm and had an infiltrative appearance **(Figure 1B)**. He was referred to our Urology department for further evaluation. CT urography, performed approximately 1 month after the MRI, showed further growth of the infiltrative left renal mass to 5.2 cm along with thrombus of the left renal vein **(Figure 1C)**. The thrombus did not extend into the IVC. Extent of disease work up including chest radiograph and serum chemistry revealed no evidence of metastasis. Serum creatinine was 1.4 mg/dL with normal liver function tests and alkaline phosphatase. Comorbidities included hypertension, type 2 diabetes and hyperlipidemia. On physical examination, there was no suspicious distant lymphadenopathy.

The patient subsequently underwent left radical nephrectomy through an open subcostal incision. A limited regional para-aortic lymphadenectomy was also performed due to small palpable lymph nodes noted at the time of surgery. Pathology showed a 7.8 cm papillary type 2, grade 3 RCC with invasion into the renal vein and negative surgical margins. The adrenal gland and a left perihilar lymph node were negative for carcinoma. Pathologic stage was pT3aN0. The patient’s postoperative course was unremarkable with a three day hospital stay. He was discharged in stable condition.

**Figure 2.**
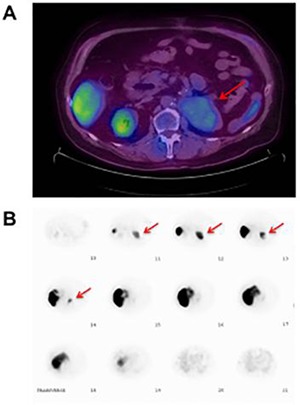
**A.** Axial fused PET/CT image demonstrating an 8.5 x 6.5 cm soft tissue mass in the left renal fossa (red arrow) with mild FDG uptake. There is physiologic FDG uptake in the liver, bowel, and right kidney. **B.** Transverse Tc-99m sulfur colloid scintigram showing uptake by the liver and spleen (red arrow), with the latter located in the left renal fossa.

Follow-up fluorine-18-fluorodeoxyglucose (FDG) PET/CT approximately 3 months following surgery demonstrated an 8.5 x 6.5 cm soft tissue mass in the left renal fossa with mild F-18-FDG uptake (SUV max of 2, with a background of 1.5) **(Figure 2A)**. Differential diagnoses included recurrent/residual disease versus post-surgical change. There was no other areas concerning for metastatic disease. The corresponding clinical presentation at this time was unremarkable; the patient had no hematuria, flank pain or any major complaints. The patient was also regaining his appetite. On examination, his lungs were clear to auscultation, abdomen was soft, non-tender and non-distended; no masses were appreciated. There were no signs of lymphadenopathy.

A review of the patient’s prior imaging and surgical history revealed the presence of a spleen on the pre-operative imaging studies and no splenectomy at the time of left nephrectomy. This raised the possibility that the soft tissue mass in question may represent the spleen that had fallen into the renal fossa. As CT and MRI scans were equivocal in this case, we decided to use radio-nucleotide imaging to further identify the mass before proceeding with any interventional procedure. A technetium-99m sulfur colloid scan was then performed, confirming the location of the spleen in the left renal fossa **(Figure 2B)**.

## Discussion

Local regional recurrence of RCC post-nephrectomy most often occurs within three years after surgery. Metastatic disease to the adrenal gland, perilymphatics and perinephric fat may be the sources of recurrence in the renal fossa. Isolated local recurrence following nephrectomy is uncommon, occurring in less than 2% at 5 years (4). Post-nephrectomy, many processes mimicking RCC recurrence have been previously described in adult patients (5–9). Page et al. reported a case of mass detected after nephrectomy and total splenectomy, which was removed due to suspicion of tumor (6). It later turned out to be accessory spleen on histological examination. Although uncommon, post-operative changes can also look like tumor. Tolhurst et al. reported a case of benign organizing hematoma mimicking recurrence of RCC (8). Besides these, there are few other reports of accessory spleen mimicking a renal tumor, where CT scans used for oncologic follow-up were unable to definitively differentiate between recurrence and other mass-occupying processes (5–9).

Our case report is unique from previously published literature, as displacement of the entire spleen into the renal fossa post-nephrectomy has not been reported in adult patients. It is evident that previous surgery, atypical location and abnormal shape make it difficult to differentiate between spleen and tumor recurrence using CT scan or MRI. Technetium-99m sulfur colloid scan is more sensitive in detecting spleen, making it useful in cases where other imaging modalities show equivocal results.

## Summary

We suggest that with a high index of suspicion, the use of the technetium-99m sulfur colloid scan would allow the characterization of these lesions as splenic in origin and prevent subjecting patients to risks of biopsy or even surgery.
